# Cryptorchidism and Testicular Tumor: Comprehensive Analysis of Common Clinical Features and Search of SNVs in the *KIT* and *AR* Genes

**DOI:** 10.3389/fcell.2020.00762

**Published:** 2020-08-07

**Authors:** Daniel Adrian Landero-Huerta, Rosa María Vigueras-Villaseñor, Emiy Yokoyama-Rebollar, Fabiola García-Andrade, Julio César Rojas-Castañeda, Luis Alonso Herrera-Montalvo, José Díaz-Chávez, Isidro Xavier Pérez-Añorve, Elena Aréchaga-Ocampo, Margarita Dolores Chávez-Saldaña

**Affiliations:** ^1^Laboratorio de Biología de la Reproducción, Instituto Nacional de Pediatría, Mexico City, Mexico; ^2^Posgrado en Ciencias Naturales e Ingeniería, Unidad Cuajimalpa, Universidad Autónoma Metropolitana, Mexico City, Mexico; ^3^Departamento de Genética Humana, Instituto Nacional de Pediatría, Mexico City, Mexico; ^4^Unidad de Investigación Biomédica en Cáncer, Instituto Nacional de Cancerología-Instituto de Investigaciones Biomédicas, Universidad Nacional Autónoma de México, Mexico City, Mexico; ^5^Instituto Nacional de Medicina Genómica, Mexico City, Mexico; ^6^Instituto Nacional de Cancerología, Mexico City, Mexico; ^7^Departamento de Ciencias Naturales, Unidad Cuajimalpa, Universidad Autónoma Metropolitana, Mexico City, Mexico

**Keywords:** SNVs, *KIT*, *AR*, isolated cryptorchidism, testicular germ cell tumor

## Abstract

Allelic variants in genes implicated in the development of testicular germ cell tumor (TGCT) could be present in patients with cryptorchidism (CO). Currently; the mechanisms explaining this relationship are still unknown. In this study the common clinical features in patients with CO and TGCT and 6 variants of *KIT* and *AR* genes associated to TGCT were analyzed. Population analyzed included 328 individuals: 91 patients with CO; 79 with TGCT, 13 of them with previous CO diagnosis, and 158 healthy males. Of the 13 patients with TGCT and history of CO, one patient (7.7%) presented the heterozygous form of the variant rs121913507 and two patients (15.4%) presented homozygote genotype for the variant rs121913506 in *KIT* gene. Interestingly, the heterozygous form for the variant rs121913506 of *KIT* gene was identifying in all of 13 patients. The rs201934623, rs774171864, and rs12014709 variants of the *AR* gene did not show any clinical association. Our results strongly support that genetic component in CO could be conditioning for the development of TGCT. Notably, *KIT* gene variants might be determinants in the pathological association between TGCT and CO.

## Introduction

Cryptorchidism (CO) or undescended testis (OMIM#219050), is the most common genitourinary malformation in newborn males. Many reports indicate that boys with CO have an overall relative risk (RR) of 4.8 (95% confidence interval 4.0–5.7) of develop testicular germ cell tumors (TGCT, OMIM#273300; Dieckmann and Pichlmeier, 2004; Kratz et al., 2010), which is the most frequent solid tumor in men between 15 and 44 years of age. TGCT is classified generally as seminoma (sTGCT) and non-seminoma (nsTGCT; Kratz et al., 2010). Although the association of CO and TGCT has been clinically established, the mechanisms leading to carcinogenesis are still unknown (Ferguson and Agoulnik, 2013). Due to the presence of common genetic factors in the etiology of both pathologies, it is possible infer the existence of a molecular genetic relationship between CO and the development of TGCT (Vigueras-Villaseñor et al., 2015).

Studies that have been focused on searching diagnostic markers for TGCT proposed several single nucleotide variants (SNVs) in genes such as *POU5F1*, *DND1*, *KIT*, *KITLG*, *AR*, *DMTR1*, *SPRY4*, *BCL2*, *NANOG*, *TGFBR2*, *PTEN*, *AKT1*, *PDE11A*, *GATA4*, and *THOC1* (Dalgaard et al., 2011; Turnbull and Rahman, 2011; Landero-Huerta et al., 2017; Litchfield et al., 2017). Proto-oncogene tyrosine kinase receptor (*KIT*) and the androgen receptor (*AR*) are relevant in the development of TGCT. Although it has not been shown that genetic variants in these genes are responsible for causing CO or Germ cell neoplasia *in situ* (*GCNIS*) a precursor lesion to TGCT, both genes have a role in testicle development and testicular carcinogenic process, influencing directly as in the case of *AR*, through non-genomic pathways (Walker, 2003). The *KIT* gene codifies a class III homodimeric receptor with tyrosine kinase activity in humans. KIT is activates by its ligand KITLG codified by the *KITLG* gene (Agarwal et al., 2014). Both, receptor and ligand are essential for survival, migration and differentiation of the early germ cells (gonocytes; Sheikine et al., 2012). Currently, gonocytes have been proposed as responsible for the development of *GCNIS* (Vigueras-Villaseñor et al., 2015; Berney et al., 2016). On the other hand, the *AR* gene produces a homodimeric cytosolic nuclear receptor, which binds to androgens and induces gene transcription (Li and Al-Azzawi, 2009; Davis-Dao et al., 2011). AR is essential in the inguinal-scrotal phase of testicular descent during the male fetal stage by controlling the normal gonocyte proliferation in the testis (Merlet et al., 2007; Hutson et al., 2015). Interestingly, AR is overexpressed in *GCNIS* and it has been found in gonocytes unable to differentiate properly (Merlet et al., 2007).

Although several studies clearly show a high risk of develop testicular neoplasia in patients with CO, there are no studies that correlate SNVs in patients with TGCT and CO. Therefore, the aims of this study were to analyze the relevant common clinical features in Mexican patients with isolated CO and TGCT, and to identify SNVs in the *KIT* and *AR* genes by allelic discrimination in patients with TGCT and history of CO. This study reports clinical features and genetic variant that may support the early diagnosis of TGCT in pediatric patients with isolated CO.

## Materials and Methods

### Subjects

The study included 328 individuals referred to the Urology Service of the National Institute of Pediatrics and National Institute of Cancerology in Mexico, from 2006 to 2017. The patients were divided in two groups as follows: the first group consisted in 91 patients with confirmed diagnosis of isolated or non-syndromic CO; the second group included 79 patients with confirmed diagnosis of TGCT, 66 of them without history of CO, and 13 patients with confirmed history of isolated CO. In all patients the diagnosis were validated by clinical history, physical examination and imaging studies in all cases. Subsequently, clinical data was obtained and patients were classified according to clinical features. In addition, 158 healthy men without history of CO or TGCT were included as a control group.

All patients and healthy men were Mexican descent, at least two generations, with 46, XY normal karyotype. This study is part of the project with registration number INP-01/2016, approved by the Research and Ethic Committee of INP and all patients included in the study had previously signed the informed consent.

### Genotyping of Allelic Variants

DNA was obtained from peripheral blood sample from patients with CO, patients with TGCT without history of CO and healthy individuals according to standard protocols QIA-AMP DNA blood mini kit, [Qiagen, Vienna, Austria]. DNA from patients with both conditions (TGCT and history of CO) was obtained from the testicular tumor samples embedded in paraffin a according to the manufacturer’s protocol FFPE RNA/DNA Purification Plus Kit, [Norgen Biotec Corp, Ontario, Canada]. Subsequently, DNA samples were used for genotyping analysis of rs121913507 (D816V), rs121913506 (D816H), and rs121913514 (N822K) SNVs in the *KIT* gene and rs201934623 (P392S), rs774171864 (A299T), and rs12014709 (g.67718624T > G) SNVs in the *AR* gene. The genotyping was performed through allelic discrimination according to established protocol of TaqMan [BMG chemistry Applied Biosystems, Foster City, CA, United States]. The genotyping rate was 99.9% and 30% of the randomized samples, which showed 100% reproducibility in duplicate trials for the 6 SNVs.

### Statistical Analysis

The comparison between the genotypes obtained and the clinical data was performed using SPSS v21 statistical package. Chi-square test or Fisher’s exact test were used for the SNVs analysis in different groups; in all cases, the *P* value < 0.05 was considered significant. The statistical power was calculated from the frequency of the minor allele of the variants with a significant *P* value due to the small number of individuals included in this study. Finally, the allelic frequencies of the SNVs in the population were compared with the frequencies reported in other populations in the HapMap and in the project of the 1000 genomes of European Ancestry (EUR), African Ancestry (AFR), East Asian Ancestry (EAS), South Asian Ancestry (SAS), Latino Ancestry (AMR), and Mexican Ancestry in Los Angeles (MXL).

### Analysis of Linkage Disequilibrium and Haplotypes

Haplotype association analysis was performed with the variants located in the same gene. Those variants with a *D*’ value equal or greater than 0.8 were considered in linkage disequilibrium (LD) and haplotypes formed by our cases and controls were compared using Haploview 4.2 software (Barrett et al., 2004), and then correlating only the patients with bilateral and unilateral CO phenotypes. The epistasis between variants located in different loci were assayed by a Multifactor Dimensionality Reduction (MDR) method in the MDR 3.0.2 software (Moore et al., 2006), since all *P* values were greater than 0.05 and Cross Validation (CV) values equal to 10/10, were considered.

### Analysis of Population Structure

The analysis of rs121913506 (D816H), rs121913514, rs774171864, and rs12014709 SNVs in 328 individuals were performed. The software STRUCTURE 2.3.4. (Pritchard Lab, Stanford University) was used to test the stratification within the samples. A mixing model consisting of a burning period of 1,000,000 and 1,000,000 repetitions with a *k* = 2 was used. It should be noted that, although the ancestry informative markers (AIMS) analysis was not performed, the SNVs allowed us to evidence the structural phenomenon of our population. The value of δ > 1 indicated that majority of the analyzed individuals were mixed (Rosenberg, 2002).

## Results

### Clinical Features Associated to CO and TGCT

At the time of diagnosis, the patients with CO (*n* = 91) had an average age of 3.5 ± 0.3 years, and those with TGCT had 26.5 ± 0.9 years [TGCT with CO (*n* = 13) = 24.3 ± 2.5 years, and TGCT without CO (*n* = 66) = 26.9 ± 1.0 years].

In patients with CO, the frequency of bilateral CO was 48.3% (44/91); all patients with CO underwent orchidopexy, but 73.6% (68/91) underwent orchidopexy after 18 months of age (2–16 years; data not shown), and only 16.5% (15/91) required orchiectomy due to the presence of testicular atrophy (Table 1). In addition, 13.2% (12/91) of the patients reported family history of CO and only 2.2% (2/91) reported family history of TGCT (Table 1).

**TABLE 1:** Clinical features of the patients.

Clinical features		Patients with isolated CO *n* = 91 (%)	Patients with TGCT *n* = 79	
			
			TGCT with CO *n* = 13 (%)	TGCT without CO *n* = 66 (%)
Age at diagnosis		3.5 ± 0.3	24.3 ± 2.5	26.9 ± 1.0
CO	Bilateral	44 (48.3)	7 (53.8)	NA
	Left	25 (27.5)	3 (23.1)	
	Right	22 (24.2)	3 (23.1)	
Orchidopexy	Yes	91 (100)	5 (38.5)	NA
	No	0 (0)	8 (61.5)	
Orchiectomy	Yes	15 (16.5)	13 (100)	66 (100)
	No	76 (83.5)	0 (0)	0 (0)
Family history of CO	Positive	12 (13.2)	0 (0)	0 (0)
	Negative	79 (86.8)	13 (100)	66 (100)
Family history of TGCT	Positive	2 (2.2)	0 (0)	3 (4.5)
	Negative	89 (97.8)	13 (100)	63 (95.5)
TGCT	Bilateral	NA	1 (7.7)	0 (0)
	Left		4 (30.8)	36 (54.5)
	Right		8 (61.5)	30 (45.5)
Clinical stage at diagnosis	I	NA	4 (30.8)	33 (50)
	II		7 (53.8)	15 (21.7)
	III		1 (7.7)	12 (18.2)
	IV		1 (7.7)	6 (9.1)
Metastasis	Positive	NA	7 (53.8)	24 (36.4)
	Negative		6 (46.2)	42 (63.6)
Tumor histological type	nsTGCT	NA	10 (76.9)	38 (57.6)
	sTGCT		3 (23.1)	28 (42.4)

*nsTGCT, Non-Seminomatous Testicular Germ Cells Tumor; sTGCT, Seminomatous Testicular Germ Cells Tumor; and NA, Not Apply.*

In the group of TGCT patients, 16.5% (13/79) had CO, of them, 38.5% (5/13) underwent orchidopexy in an average of 8.8 years of age. All patients of the TGCT group (79/79) underwent orchiectomy at TGCT diagnosis. The most frequent histological type of TGCT patients was nsTGCT [61% (48/79)]. The subgroup of 13 patients with TGCT and CO did not report family history of CO or TGCT, while in the subgroup of TGCT without CO, only 4.5% (3/66) patients had family history of TGCT (Table 1).

### Genotyping

In order to identify a genetic marker associated to CO condition or to genetic susceptibility to develop TGCT in patients with CO, 6 SNVs were analyzed in this study. Three SNVs in the *KIT* gene, including rs121913507 (D816V), rs121913506 (D816H), and rs121913514 (N822K) were evaluated. While the 3 SNVs analyzed in the *AR* gene were rs201934623 (P392S), rs774171864 (A299T), and rs12014709 (c.66718624 T/G). Initially the subgroups of patients with TGCT and history of CO (*n* = 13) were compared with those patients with TGCT without CO (*n* = 66). Posteriorly, we compared the subgroup of TGCT and CO patients with isolated CO patients (*n* = 91), and finally, with healthy controls (*n* = 158; Table 2). Results showed that the variant rs121913507 (D816V) of the *KIT* gene was found in 1/13 (7.7%) of patients with TGCT and CO in a heterozygous form, while the rest of patients [12/13 (92.3%)] presented a homozygote genotype (AA). The comparison among the rest of the patients and healthy controls did not show significant statistically differences [TGCT + CO *vs* TGCT (*P* = 0.162), TGCT + CO *vs* CO (*P* = 0.125), and TGCT + CO *vs* Controls (*P* = 0.076), respectively]. The variant rs121913506 (D816H) of the *KIT* gene was found in heterozygous form in 11/13 (84.6%) patients with TGCT and CO, while 2/13 (15.4%) patients with TGCT and CO had a homozygote genotype (CC). In contrast to the variant rs121913507 (D816V), the comparison between the variant rs121913506 (D816H) of patients with TGCT and CO with the rest of the patients and healthy controls showed statistically significant differences [TGCT + CO *vs* TGCT (*P* = 0.025), TGCT + CO *vs* CO (*P* = 0.015), and TGCT + CO *vs* Controls (*P* = 0.005), respectively]. The analysis of the rs121913514 (N822K) variant of the *KIT* gene between all groups did not show any differences (Table 2).

**TABLE 2:** Genotypic frequency of the allelic variants in *KIT* and *AR* genes in Mexican population.

SNV	Patients with TGCT and CO *n* (%)	Patients with TGCT *n* (%)	*P* value TGCT + CO *vs* TGCT	Patients with CO *n* (%)	*P* value TGCT + CO *vs* CO	Healthy Controls *n* (%)	*P* value TGCT + CO *vs* Control
*KIT* GENE							
D816V (A/T) rs121913507 c.54733155	AA = 12 (92.3) AT = 1 (7.7) TT = 0 (0)	AA = 66 (100) AT = 0 (0) TT = 0 (0)	0.162	AA = 91 (100) AT = 0 (0) TT = 0 (0)	0.125	AA = 158 (100) AT = 0 (0) TT = 0 (0)	0.076
D816H (G/C) rs121913506 c.54733154	GG = 0 (0) GC = 11 (84.6) CC = 2 (15.4)	GG = 0 (0) GC = 66 (100) CC = 0 (0)	0.025	GG = 0 (0) GC = 91 (100) CC = 0 (0)	0.015	GG = 0 (0) GC = 158 (100) CC = 0 (0)	0.005
N822K (A/T) rs121913514 c.54733174	AA = 0 (0) AT = 13 (100) TT = 0 (0)	AA = 0 (0) AT = 66 (100) TT = 0 (0)	NA	AA = 0 (0) AT = 89 (97.8) TT = 2 (2.2)	0.765	AA = 0 (0) AT = 154 (97.5) TT = 4 (2.5)	0.693
*AR* GENE							
Intronic (T/G) rs12014709 c.66718624	TT = 0 (0) TG = 13 (100) GG = 0 (0)	TT = 2 (3.0) TG = 63 (95.5) GG = 1 (1.5)	0.736	TT = 4 (2.5) TG = 80 (87.9) GG = 7 (7.7)	0.415	TT = 4 (2.5) TG = 143 (90.5) GG = 11 (7.0)	0.508
P392S (C/T) rs201934623 c.67546320	CC = 13 (100) CT = 0 (0) TT = 0 (0)	CC = 66 (100) CT = 0 (0) TT = 0 (0)	NA	CC = 91 (100) CT = 0 (0) TT = 0 (0)	NA	CC = 158 (100) CT = 0 (0) TT = 0 (0)	NA
A299T (C/T) rs774171864 c.67546042	CC = 0 (0) CT = 13 (100) TT = 0 (0)	CC = 0 (0) CT = 66 (100) TT = 0 (0)	NA	CC = 0 (0) CT = 91 (100) TT = 0 (0)	NA	CC = 0 (0) CT = 158 (100) TT = 0 (0)	NA

*NA = Not Apply.*

The results of the analysis of the SNVs in the *AR* gene showed that the rs12014709 (g.67718624T > G) variant located in the intronic region of gene presented different genotypes, however, no statistically significant differences resulted between the groups (Table 2). The variants rs201934623 (P392S) and rs774171864 (A299T) of the *AR* gene did not show any differences.

### TGCT With History of CO Patients and the *KIT* Gene

Considering that the group of patients with TGCT and CO showed significant differences in the genotypes of the *KIT* gene, we analyzed their association with significant clinical features (Table 3). In the first analysis, the patients 2 and 3 that presented the variant rs121913506 (D816H) in homozygous form, were young and suffered unilateral CO (although in opposite side), however, both presented different tumor histological type and only one of them developed metastasis. By the other hand, the patient 13 presented the variant rs121913507 (D816V) in heterozygous form, he had history of bilateral CO and TGCT was unilateral, the tumor type was nsTGCT and he did not develop metastasis.

**TABLE 3:** Clinical features of the patients with TGCT and isolated CO.

Patient	Age at diagnosis of TGCT	CO side	Age of orchidopexy	Tumor side	Tumor histological type	Metastasis	Clinical stage at diagnosis	Genotype

	Patients with orchidopexy							
1	16 years	Bilateral	8 years	Right	nsTGCT	No	I	
2	18 years	Right	8 years	Right	sTGCT	No	I	D816H/D816H
3	19 years	Left	12 years	Left	nsTGCT	Retroperitoneum	II	D816H/D816H
4	24 years	Right	10 years	Right	nsTGCT	No	I	
5	27 years	Bilateral	6 years	Right	sTGCT	No	I	

	**Patients without orchidopexy**							

6	16 years	Bilateral	NA	Right	nsTGCT	Retroperitoneum	II	
7	17 years	Bilateral	NA	Left	nsTGCT	Pelvis	II	
8	22 years	Left	NA	Left	sTGCT	Retroperitoneum	II	
9	22 years	Bilateral	NA	Bilateral	nsTGCT	No	II	
10	23 years	Right	NA	Right	nsTGCT	Lung	IV	
11	27 years	Left	NA	Left	nsTGCT	Retroperitoneum	II	
12	36 years	Bilateral	NA	Right	nsTGCT	Lung	III	
13	49 years	Bilateral	NA	Right	nsTGCT	No	II	Wild type/D816V

*NA = Not Apply.*

Interestingly, higher frequency of metastasis was observed in patients with TGCT and previous CO (*n* = 13) compared to those who presented only TGCT without history of CO (*n* = 66) [53.8% (7/13) *vs* 36.4% (24/66)] and histological type of nsTGCT [76.9% (10/13) *vs* 57.6% (38/66)] (Table 1). Furthermore, only 38.5% (5/13) of patients with TGCT and previous CO underwent orchidopexy in an age average of 8.8 years, while the rest [8/13 (61.5%)] of these patients did not have orchidopexy. The nsTGCT histological type [87.5% (7/8) *vs* 60% (3/5)], metastasis [75% (6/8) *vs* 20% (1/5)], and more advanced tumor stage were most frequently presented in these 8 patients that did not underwent orchidopexy (Table 3).

### Allelic Frequencies

The allelic frequencies for the 3 variants of the *KIT* gene and 3 variants of the *AR* gene analyzed in this study were search in the literature and in different worldwide databases for general population, to compare these information to the allelic frequencies observed in our results. Only the variants rs121913507, rs201934623, rs774171864, and rs12014709 coincided with the reported frequencies (Table 4).

**TABLE 4:** Allelic frequencies of the variables analyzed in the population studied.

Gene	SNV	Allele	ALL	EUR	AFR	EAS	SAS	AMR	MXL	Mexican
*KIT*	rs121913507 D816V	A	1	1	1	1	1	1	1	1
		T	0	0	0	0	0	0	0	0
	rs121913506 D816H	G	—	—	—	—	—	—	—	0.50
		C	—	—	—	—	—	—	—	0.50
	rs121913514 N822K	A	—	—	—	—	—	—	—	0.49
		T	—	—	—	—	—	—	—	0.51
*AR*	rs201934623 P392S	C	0.99	1	1	1	0.96	1	0.99	1
		T	0.01	0	0	0	0.04	0	0.01	0
	rs774171864 A299T	C	1	1	1	1	1	1	1	0.50
		T	0	0	0	0	0	0	0	0.50
	rs12014709 Intronic	T	0.85	0.91	0.58	1	0.96	0.91	0.96	0.49
		G	0.15	0.09	0.42	0	0.04	0.09	0.04	0.51

*There are no data available on the frequencies of the variant (—), ALL, All gnomeAD, EUR, European Ancestry, AFR, African Ancestry, EAS, East Asian Ancestry, SAS, South Asian Ancestry, AMR, Latino Ancestry, and MXL, Mexican Ancestry in Los Angeles.*

Haplotype blocks conformed by variants located in the same gene were not identify. In the same way, MDR analysis did not show any significant statistical gene-gene interaction. However, the analysis of the population structure of the 4 of the 6 SNVs, a value of δ = 2.27 was identified.

## Discussion

Despite the high rate of spontaneous descent during the first year of life, CO is still one of the most common congenital malformations among males worldwide, with a frequency of 1–3%; and is one of the risk factors for development TGCT (Banks et al., 2013). Besides, TGCT is the most common cancer in young men between 15 to 44 years (Trabert et al., 2014), with an age-adjusted rate of 11.1 per 100,000 men (world standard). Among the solid tumors with higher mortality, TGCT are found in the fifth place with a mortality rate of 1.3 per 100,000 men (Global Cancer Observatory, 2020). In spite of clinical relationship between CO and TGCT the molecular mechanisms underlying both diseases are still unknown. Significantly, although the incidence of CO and TGCT has increased worldwide, in Mexico there are few epidemiological data about both diseases.

In this study, we analyzed the clinical manifestations of patients with CO, patients with TGCT and patients with TCGT and history of CO. In the group of patients with CO we found that the incidence of bilateral disease was higher than those reported for Hispanic population [48.3% (44/91) *vs* 19.7% (25/127)] (Davis-Dao et al., 2012). However, other findings were similar to other studies. Surgical intervention in 70% of the patients with CO done after 2 years (Williams et al., 2018) compared with 73.6% in our study, and the family history of TGCT in our patients with CO and in Italian population [2.2% (2/91) *vs* 2.1% (15/721; Foresta et al., 2008)] (Table 1).

In addition, in the group of TGCT patients we did not find CO history, compared to other reports who found CO history in 5–10% (Kratz et al., 2010) and 14.6% (18/123) of TGCT patients (Garolla et al., 2005). On the other hand, we identify 4.5% patients with TGCT without CO (3/66) with at least one family member affected by the same neoplasm, and a global frequency of 3.8% (3/79 patients with TGCT), these were according to other authors for familiar TGCT (Mai et al., 2009; Rapley and Nathanson, 2010); we also observed higher frequency of nsTGCT in our patients with TGCT, similar to Hispanic population (Woldu et al., 2018; Table 1).

CO is an important risk factor for the development of TGCT, and this relationship has been clinically established, however, the molecular mechanism between failure in testicular descent and the development of malignancy, is still unknown (Ferguson and Agoulnik, 2013). Several genes have been associated to the development of TGCT (Rapley and Nathanson, 2010; Landero-Huerta et al., 2017), but only a few of them, such as *KIT* gene expression (Vigueras-Villaseñor et al., 2015), or polyQ inserts in exon 1 of the *AR* gene (Ferlin et al., 2005; Davis-Dao et al., 2011; Hutson et al., 2015; Fukawa and Kanayama, 2018) have been analyzed in patients with CO, without establish a relationship between CO and TGCT.

Therefore, we focused on determine the relationship between 6 SNVs in both *KIT* and *AR* genes and TGCT and CO in Mexican population. The contrast of the patients with TGCT and history of CO with TGCT without CO, or those with CO as well as control group showed statistical differences in the presence of the homozygous form (CC) of the rs121913506 (D816H) variant of the *KIT* gene in two patients with TGCT and history of CO, these results suggested that CO patients with C allele in homozygous form might have a higher risk of development TGCT. The frequency of C allele in homozygous form in our group of TGCT Mexican patients was similar to the reported in Japanese population (Sakuma et al., 2003) [2.5% (2/79) *vs* 2.9% (1/34)]. Although the other SNVs did not show statistical differences, we consider pertinent not to discard them as important SNVs in Mexican population, therefore, these results must be explored in higher number of samples. In previous reports, the rs121913506 (D816H) variant has been considered as mutation related to TGCT (Looijenga et al., 2003), however, we identified the same variant in heterozygous form in the majority of patients and even in the control group, therefore our population could have a different genotype.

In the group of patients with TGCT, we considered the simultaneous presence of TGCT and CO as the most important factor, so we analyzed individually this subgroup of 13 patients, and we found that the variants rs121913507 (D816V) and rs121913506 (D816H) of the *KIT* gene were identified in 3 patients (2, 3, and 13 patients; Table 3); however, there were not association between these genetic variants and any particular phenotype or clinical feature. However, the results obtained from the clinical characteristics indicated that the patients with TGCT with previous CO could express a more aggressive phenotype of the TGCT, which itself is already severe (Table 3). These results suggest that the relationship between CO and severe TGCT phenotypes could be due to the participation of the function of multiple genes and not exclusively by *KIT* and *AR*. In particular, we observed that patients who did not undergo orchidopexy at right time had a more severe TGCT phenotype (Table 3). We could highlight the importance of performing orchidopexy in a timely manner, minimizing the risk and complications in the pediatric patient with CO (Table 3), and avoiding undescended testes to be subject to different abnormal stress conditions (Williams et al., 2018).

Additionally, the comparison of the allelic frequencies for the rs121913506 (D816H) and rs121913514 (N822K) variants in other populations was not possible due to lack of available information in the HapMap and in the project of the 1000 genomes. However, the frequencies for the allele A of the variant rs121913507 (D816V) in the *KIT* gene, and for the allele C of the variant rs201934623 (P392S) in the *AR* gene, were similar to that found in all populations. In the case of the both alleles of the variant rs774171864 (A299T), the frequencies were different to all databases, and the allelic frequencies of the variant rs12014709 were similar only with the AFR. Finally, we identified a mixed population in population structure analysis, according to previously reports for Mexican population with predominantly Amerindian and EUR (Salazar-Flores et al., 2015).

In conclusion, the results of this study support the fact that CO is a risk factor for the development of TGCT at molecular level. *KIT* gene variants rs121913507 and rs121913506 might be common among TGCT and CO. However, more studies must perform to clarify these results and to find predictive biomarkers for TGCT and CO patients.

## Data Availability Statement

The raw data supporting the conclusions of this article will be made available by the authors, without undue reservation, to any qualified researcher.

## Ethics Statement

The studies involving human participants were reviewed and approved by the project with registration number INP-01/2016, approved by the Research and Ethics Committee of INP. Written informed consent to participate in this study was provided by the participants’ legal guardian/next of kin.

## Author Contributions

MC-S contributed to conception and design. DL-H, MC-S, FG-A, and JR-C contributed to the development of the methodology. MC-S, RV-V, EY-R, IP-A, and EA-O performed the analysis and interpreted the data. DL-H, MC-S, and RV-V contributed to writing, review, and revision of the manuscript. LH-M, JD-C, and JR-C provided administrative, technical, or material support. RV-V, MC-S, and DL-H contributed to study supervision. All authors contributed to the article and approved the submitted version.

## Conflict of Interest

The authors declare that the research was conducted in the absence of any commercial or financial relationships that could be construed as a potential conflict of interest.
